# Animals as an indicator of carbon sequestration and valuable landscapes

**DOI:** 10.3897/zookeys.100.1547

**Published:** 2011-05-20

**Authors:** Jan Szyszko, Axel Schwerk, Jarosław Malczyk

**Affiliations:** Laboratory of Evaluation and Assessment of Natural Resources, Warsaw University of Life Sciences, Nowoursynowska Street 166, 02-787 Warsaw, Poland

**Keywords:** Carabidae, indicators, landscape evaluation, Mean Individual Biomass (MIB)

## Abstract

Possibilities of the assessment of a landscape with the use of succession development stages, monitored with the value of the Mean Individual Biomass (MIB) of carabid beetles and the occurrence of bird species are discussed on the basis of an example from Poland. Higher variability of the MIB value in space signifies a greater biodiversity. Apart from the variability of MIB, it is suggested to adopt the occurrence of the following animals as indicators, (in the order of importance), representing underlying valuable landscapes: black stork, lesser spotted eagle, white-tailed eagle, wolf, crane and white stork. The higher number of these species and their greater density indicate a higher value of the landscape for biodiversity and ecosystem services, especially carbon sequestration. All these indicators may be useful to assess measures for sustainable land use.

## Animals, carbon sequestration and landscapes

As part of the ongoing discussion on the sustainable use of landscapes and ecosystem services we give here an overview on the relationship between biodiversity and carbon sequestration in forests as revealed by a long-term study in north-western Poland with particular reference to carabid beetle diversity.

From the very beginning of life, environmental resources on Earth have been shaped by natural succession processes, influenced by climate, and by various disturbances such as orogenic movements, windfall, floods and fires. Depending on latitude and the geological base, these disturbance factors vary among various places on the globe. The lack of catastrophes entails the buildup of organic substances, which can be measured simply with the content of carbon. The content of carbon in a single hectare of natural forest of the moderate climatic zone shaped by succession processes for thousands of years, exceeds 350 tons/ha with upper limits estimated between 500 and 700 tons/ha (Luyssaert et al. 2008). Half of this resource is stored in living organisms (plants, animals, fungi) while the other half is stored in the soil (litter and humus compounds in the mineral soil) (e.g. Oak Ridge National Laboratory 2010).

**Figure 1.**
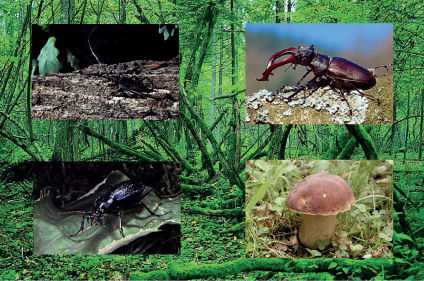
A natural forest with a carbon content within the limit of 350 tons per ha and a mean individual biomass (MIB) for carabid beetles exceeding 350 mg and with species characteristic for that environmental system. *Lucanus cervus* – a species linked to old decaying oak wood (top right), *Cerambyx cerdo* – a species linked to living old oaks (top left), *Boletus erythropus* – a mycorrhizal species occurring in soils with a historically well developed soil profile (bottom right), *Carabus intricatus* – a species occurring in old forest environmental systems with an easily decomposing duff (bottom left).

**Figure 2.**
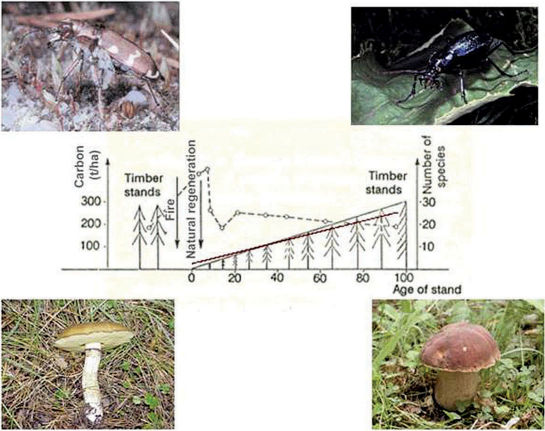
Succession of carbon content (left y-axis and black line) and species diversity (right y-axis and striped line) in a stand after the destruction of the trees by fire. After the fire, the carbon content is low with *Cicindela hybrida* and the sticky bun (*Suillus luteus*) as characteristic species (left photographs). In 100 years old stands the carbon content is high with *Carabus intricatus* and the dotted stem bolete *Boletus erythropus* as characteristic species (right photographs) (from Szyszko 2007).

**Figure 3.**
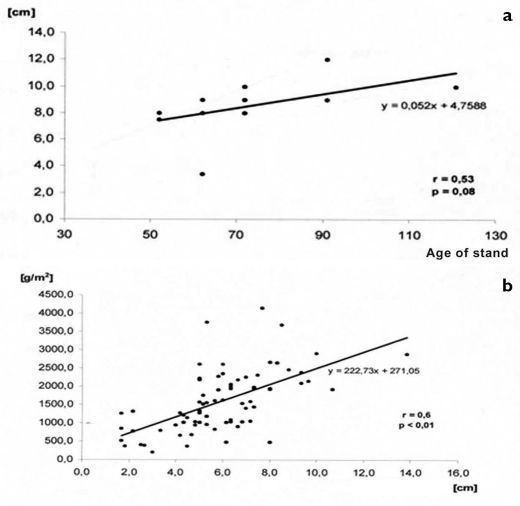
**a** Relationship between the age of a forest stand and thickness of the litter layer **b** relationship between the thickness of the litter layer and the weight of carbon per 1 sq. m (from Szyszko et al. 2003).

In such old forests (old ecosystems created by nature), ecosystems have a specific composition of plant, animal and fungal species. Species that are linked to old trees, old decaying wood, and well developed soil, occur here (Fig. 1). In such forests in the European geographic zone the full range of carabid species with a narrow geographic arrangement, low dispersal power and large body size occurs. The Mean Individual Biomass (MIB) of the carabids beetles might exceed 350 mg in such forests (Szyszko 1990, 2002). Forests of old successional stage are poor in butterflies (Szyszko 2003), the majority of bumblebees (Skrok 2003) and open-area birds (Kruszewicz 2007). With no humans present, their occurrence could only be possible due to ecological catastrophes mentioned above. Fires, floods or windfalls decelerated developmental succession processes (buildup of organic substance) and thereby impoverish these systems by releasing carbon in the form of carbon dioxide to the atmosphere (Magnani et al. 2007). Such disturbances create opportunities for species characteristic for early stages of succession, e.g., carabids species of open and low vegetation or the above mentioned butterflies, bumblebees and birds characteristic of open areas. Historically, nature itself provided advantageous conditions for the full range of biodiversity of the native fauna and flora thanks to events we call “ecological catastrophes”. These catastrophes destroy old stages of succession containing much carbon and create room for species characteristic of early successional stages (Fig. 2). It is here that species such as the nightjar (*Caprimulgus europaeus*), skylark (*Alauda arvensis*), northern dune tiger beetle (*Cicindela hybrida*) and, with the appearing pine wilding, the sticky bun (*Suillus luteus*) can strive (Fig. 2). The created open areas are also an excellent place for “landscape species”, i.e., species that need different succession stages in a wider landscape for establishment of their populations (Szyszko K. 2003, Skrok 2003). Such species breed for example in places of advanced stages of succession (natural forests with high carbon content) and hunt in open areas (early successional stages with low carbon content). The common buzzard and the majority of falconids are typical examples of such species. They nest in old trees in forests and hunt where visibility is good, i.e. in open areas or environmental systems of early successional stages, with low carbon content. Ecological catastrophes also create opportunities for many species associated with different stages of natural succession by setting back the climax situation and thereby reducing the carbon content in the environmental systems (Fig. 3).

**Figure 4.**
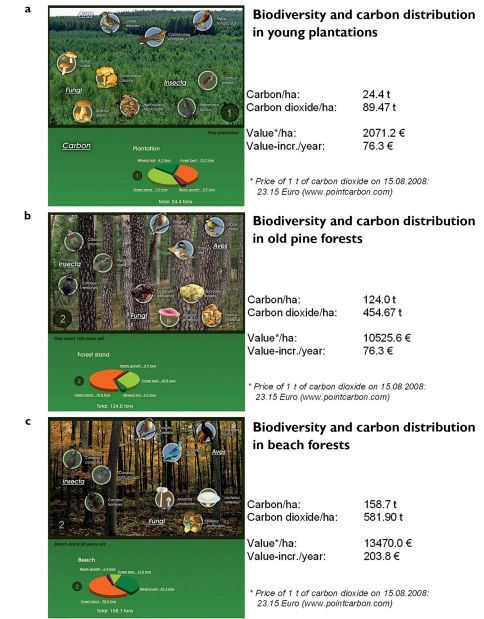
The occurrence of characteristic birds, carabid beetles and fungi as well as the structure of the carbon content in tons per ha in a forest stand, litter and mineral soil up to 10 cm in depth in a: **a** 10 year old pine stand with a MIB value of about 50 mg, created after the clear-cut of a timber pine stand (more than 100 years old) **b** ca. 60 year old pine stand with a MIB value about 250 mg. **c** ca. 80 year old beech stand created from the undergrowth after the clear-cut of a pine stand with a MIB value of about 350 mg. In all graphs the annual accumulation of carbon in that stand, the value of such accumulation and the value of the entire carbon content (forest stand + litter + mineral soil) are expressed in carbon dioxide at the prices of the European Emission Trade System on 15.08.2008 (all graphs from Szyszko 2007).

Humans play a similar role in shaping ecosystems and landscapes. Ecological catastrophes can destroy the effects of our economic activities and constitute a danger our its safety. This is why humans manage the environment by trying to reduce floods, fires and windfalls. However, to preserve the full range of biodiversity, humans replace the forces of nature and mimic its destructive role by active management. This can clearly be illustrated for forests in Poland. If it was not for the interference with natural succession and the reduction of the carbon content of up to a few dozen tons per ha (Fig. 4a) due to clearcuts, no pine cultivations could have emerged in forest areas. Consequently, bird species such as the nightjar (*Camprimulgus europaeus*) and woodlark (*Lullula arborea*) would have had no place to nest and the majority of birds of prey nesting in old trees would not have been able to hunt. Carabid beetles like *Carabus nitens*, *Bembidion nigricorne*, *Pterostichus lepidus*, *Calathus erratus*, *Masoreus wetterhallii* and *Harpalus rufitarsis* (resulting in a MIB value of about 50 mg) would not have occurred in this region (Szyszko 1990). Additionally, the mass appearance of fungal species such as sticky buns (*Suillus luteus*), sulfur tufts (*Hypholoma fasciculare*) or, slightly later, chanterelles (*Cantharellus cibarius*) and porcinis (*Boletus edulis*) would not have been possible (Fig. 4a). The destruction of forest habitats due to the felling of trees provides opportunities for the natural succession to start again, entailing a change in species composition with time. With an increase in the carbon content of a stand an increase in the MIB value of epigeic carabid beetles takes place, indicating regeneration of the environmental resources. In a ca. sixty year old pine stand (Fig. 4b), carbon content and the MIB value (about 250 mg) is higher compared to a young pine plantation (Fig. 4a). The species composition of birds, carabids and fungi differs clearly. Birds characteristic for the old pine stand include the chaffinch (*Fringilla coelebes*), great tit (*Parus major*) and coal tit (*Parus ater*). Carabids characteristic for this stage of succession include *Carabus arcensis*, *Carabus nemoralis* and *Pterostichus niger* and the most frequent and numerous fungi include the sickener (*Russula emetica*), brown roll-rim (*Paxillus involutus*), false morel (*Gyromitra esculenta*) and the cauliflower mushroom (*Sparassis crispa*). The planting of beech as undergrowth in ca. sixty year old pine stands followed by the removal of the pines ten years later results in the creation of beech stands, several of which in Poland are about eighty years old (Rylke and Szyszko 2002, Fig. 4c). When compared with a sixty year old pine stand, those old beech stands have higher carbon content and the MIB value of the carabid beetles exceeds 350 mg. Logically, characteristic species of birds, carabids and fungi are also different for the forest types (successional stages) presented above. Characteristic birds in the old beech stands are the black woodpecker (*Drycopus martius*), stock pigeon (*Columba oenas*) and chaffinch (*Fringilla coelebes*). Characteristic carabids include *Carabus coriaceus*, *Carabus hortensis* and *Carabus intricatus*. Characteristic fungi are the dotted stem bolete (*Boletus erythropus*), fleecy milk-cap (*Lactarius vellereus*) and the death cap (*Amanita phalloides*).

**Figure 5.**
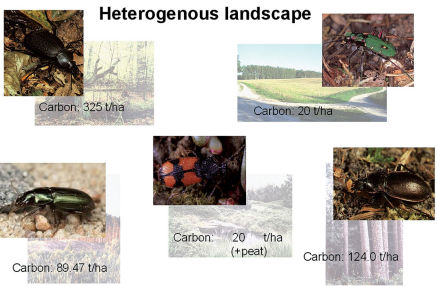
Examples of several habitats with different carbon contents and different characteristic carabid species, building a heterogenous landscape. Top left – a natural forest with a carbon content of 350 ton per ha with *Carabus coriaceus*, top right – arable land with a carbon content 20 tons per ha with *Cicindela campestris*, in the middle – a peatbog with a very high content of carbon per ha with *Panagaeus bipustulatus*, bottom left – a clear-cut with a carbon content of ca. 90 tons per ha with *Harpalus rufitarsis*, Bottom right – a timber stand with the carbon content 124 tons with *Carabus nemoralis* (from Szyszko 2007).

**Figure 6.**
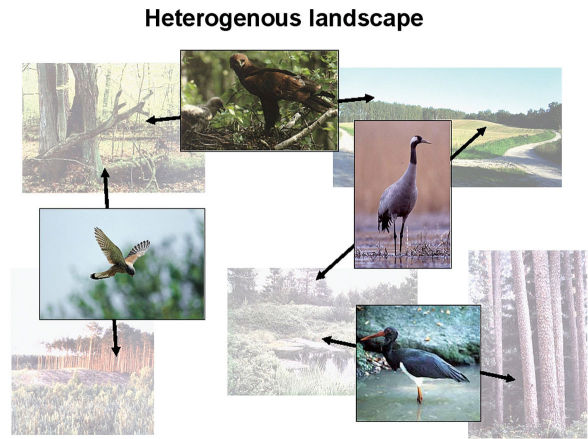
Landscape species in a heterogenous landscape. The lesser spotted eagle (*Aquila pomarina*) nests in old trees in natural and cultivated forests, and hunts in wastelands. The crane (*Grus grus*) nests in peat bogs and hunts in wastelands. The kestrel (*Falco tinnunculus*) nests in old trees in natural and cultivated forests and hunts in clear-cut areas. The black stork (*Ciconia nigra*) nests in old trees in natural and cultivated forests and hunts in peat bogs (from Szyszko 2007).

Data on different forests presented above suggest that a greater spatial differentiation of the carbon content in environmental systems, or in other words a greater differentiation of successional stages as measured using the MIB value of carabid coenoses, entails greater biodiversity (Fig. 5, Szyszko 2002). Hence, variability in space of the MIB value is a good measure for the value of the landscape (Fig. 5, see also Rylke and Szyszko 2001). However, the value of a landscape can be even more completely assessed if we also take into account the occurrence of species that use various successional stages, so called landscape species (Szyszko 2002). Typical examples of such species among birds are the black stork (*Ciconia nigra*) and the lesser spotted eagle (*Aquila pomarina*) (Fig. 6). These birds nest in old trees in vast forests, similar to natural ones, while they hunt in nearby meadows, floodplains and marshes. If these species are present, we can be sure that the said habitats occur in a given environmental complex and, what is more, they are not far apart. Species with similar requirements are the white-tailed eagle (*Haliaetus albicilla*) and the wolf (*Canis lupus*) (Szyszko 2002). The existence of both species seems to be linked to vast areas covered by both old and young successional stages. The former seem to play a significant role as localities for reproduction while the latter serves as a food acquisition area. Among these generally known and easily recognizable species, two others are worth mentioning: the crane (*Grus grus*) and white stork (*Ciconia ciconia*) (Szyszko 2002). Cranes choose old peatbogs and marshes surrounded by forests as a nesting site and gather food in meadows and fields, while the white stork nests mainly in villages surrounded by extensively used arable lands and feeds in meadows, marshes, fields and pastures. If we adopt Andrzejewski’s (1992) definition of the landscape as a “set of ecosystems linked by mutual dependencies creating an ecological system of a higher order”, we can assume that, as the occurrence of species characteristic for individual stages of succession is a measure of the advancement of these processes in biocoenoses, the occurrence of species using varied successional stages can be a measure of the quality and value of a landscape and an indicator of its functionality in space (Szyszko 2002). In light of these results, the suggestion to adopt the mentioned ‘landscape species’ as landscape value indicators has to be taken into account, with order of importance as follows: black stork, lesser spotted eagle, white tailed eagle, wolf, crane and white stork. The higher the number of these species and the greater their density, the higher the value of a landscape, in terms of environmental quality and biodiversity (Szyszko 2002).

Landscape quality includes biodiversity (highest in mosaic landscapes) and carbon storage (highest in peat bogs and old forests) and sequestration (highest in regenerating forests), as well as other ecosystem services. Sustainable land use is essential in maintaining or creating landscapes with high natural qualities. There is a need to assess measures of sustainable land use, and various animals mentioned in this paper, including carabid beetles and their MIB values, may serve as important indicators for this.
